# Trends in cannabis adverse reaction reports: A descriptive analysis of spontaneous reporting data submitted to the Canada Vigilance Program since legalization and regulation of cannabis for non-medical purposes in Canada

**DOI:** 10.1186/s42238-025-00310-x

**Published:** 2025-08-12

**Authors:** Sieara Plebon-Huff, Nadia Aziz, Marko Cavar, Safia Hassan, Maria Aoun, Shahid Perwaiz, Hanan Abramovici

**Affiliations:** Office of Cannabis Science and Surveillance, Strategic Policy Directorate, Controlled Substances and Cannabis Branch, Health Canada, 150 Tunney’s Pasture Driveway, Ottawa, ON K1A 0T6 Canada

**Keywords:** Cannabis, Real-world data, Post-market surveillance

## Abstract

**Background:**

The cannabis control framework implemented by Canada in October 2018 established a robust post-market surveillance system for cannabis products, adopting tools and practices from the existing pharmacovigilance system for pharmaceuticals and health products. The cannabis vigilance system relies on spontaneous reporting of adverse reactions, allowing Health Canada to collect, monitor and assess health effects involving cannabis. In this study, we examine trends in adverse reaction reports involving legal cannabis products since legalization and regulation in Canada.

**Methods:**

Unique case reports of adverse reactions involving cannabis were collected through the Canada Vigilance Program. Case details were extracted from each report involving legal cannabis as a suspected product. Each case was also assessed for causality to determine the likelihood of association between the cannabis product(s) and the reported event(s). The case data was then aggregated and descriptively analyzed to identify adverse reaction case patterns, including the demographic profiles and use patterns of individuals reporting adverse reactions to cannabis.

**Results:**

Overall, individuals reporting an adverse reaction to a cannabis product (n = 698) were 56.0 ± 20.0 years of age. 45.4% of reporting individuals were female, and 67.5% of individuals self-reported using cannabis for medical purposes, with pain management as the most cited reason for medical use. Most cases were reported as serious (62.3%), citing “other medically important condition” as the primary reason for seriousness (58.6%), and 68.8% of all cases involved cannabis extracts. Frequently reported events included hallucination, headache, nausea, dizziness and dyspnea. Some events were more frequently reported with products containing a greater concentration of tetrahydrocannabinol to cannabidiol, whereas others were more frequently reported with products containing a greater concentration of cannabidiol to tetrahydrocannabinol. Causality was assessed for 668 events; most were assessed as being “possibly” associated with the reported cannabis product.

**Conclusions:**

The post-market adverse reaction reporting system for cannabis products has provided valuable safety information about cannabis products available for legal retail sale in Canada. The data collected through this framework have helped identify emerging risks associated with legal cannabis products; contributed to international data about cannabis effects and risks; informed the development of communication materials related to new and emerging risks; and provided evidence to inform regulatory decisions.

## Background

On October 17, 2018, Canada became the second country in the world to federally legalize and regulate recreational cannabis following the coming into force of the *Cannabis Act* (2016). This legislative shift granted adult Canadians access to legal cannabis for non-medical use, complementing the *Access to Cannabis for Medical Purposes Regulations* (2021) which replaced the original *Marihuana Medical Access Regulations* (2022) in 2016.

In developing the *Cannabis Act*and its Regulations, the Government of Canada leveraged existing regulatory frameworks for tobacco products, foods, and health products. In line with recommendations from the Task Force on Cannabis Legalization and Regulation (2022), the Government put a system in place to provide ongoing surveillance data regarding the health consequences of cannabis use, known as the vigilance framework for cannabis. Part of this surveillance system drew on the established model of pharmacovigilance for pharmaceuticals and other regulated health products. Pharmacovigilance refers to the science and activities related to detecting, assessing, understanding, and preventing adverse reactions (also referred to as side effects) (2023). The robust pharmacovigilance system implemented for cannabis allows for the continuous monitoring of adverse reactions and emerging risks, helping to identify potential health concerns that may not have been evident at the time of legalization.

Unlike pharmaceuticals, there is no pre-market safety review for cannabis products, however the post-market surveillance system for health products was readily adaptable to cannabis, allowing Health Canada to identify, evaluate and mitigate risks associated with use of legal retail cannabis products, including adverse reactions, cannabis-drug interactions, overconsumption and risks in vulnerable populations such as children, youth, older adults, and those with underlying health conditions.

Regulatory amendments introduced in October 2019, coupled with industry innovation and shifting market dynamics have led to a rise in the diversity of available legal cannabis products, including ingestible and inhalable extracts, edibles, and topicals (2024). The increasing variety of cannabis products available in the legal market has increased the importance of monitoring the risk profile of product classes for which there is less available research. Legal cannabis products vary widely in product formulations, dosages, and methods of consumption, which may contribute to unpredictable pharmacological effects and side effects (2022, 1985).

This study aimed to describe adverse reaction reporting trends for cannabis products using data collected by Health Canada under the vigilance framework for cannabis products since the coming into force of the *Cannabis Act* and its Regulations (October 17, 2018) up to December 31, 2024.

## Methods

### Study design and setting

The data used in this study was primarily received through Health Canada’s Canada Vigilance Program (2001) and are housed in the Canada Vigilance database (2016). This database contains information about suspected adverse reactions to various regulated products, including drugs, natural health products, medical devices and cannabis. Some of the data was collected through other organized reporting systems, like complaints submitted through the Cannabis Reporting Form (2019), and uploaded to the Canada Vigilance database.

Health Canada conducts near-time monitoring, detection and assessment for cases of adverse reactions involving legal cannabis products (i.e., cannabis manufactured by a licenced producer that is packaged and labeled for retail sale) as part of the post-market vigilance framework for cannabis. Health Canada also monitors cases involving cannabis as a substance (i.e., from unknown or illegal sources) for broader issues of public health importance such as vaping-associated lung illness, pediatric poisonings, and other potential emerging safety issues. However, the present study focuses exclusively on case reports involving legal cannabis products.

Most case reports involving cannabis that are received by Health Canada are reported spontaneously by consumers, patients, health professionals, retailers or cannabis licence holders. Notably, cannabis licence holders have a regulatory obligation to submit any serious adverse reactions to Health Canada within 15 days of becoming aware (paragraph 248.1(1)(a) of the *Cannabis Regulations*). Consumers, patients, health professionals and retailers can voluntarily submit reports to Health Canada at any time. Reports may also originate from research studies, including observational studies (non-interventional, real-world), interventional human studies involving cannabis that fall outside of the regulatory definition of a clinical trial (e.g., non-therapeutic research on cannabis studies), other organized data collection systems (e.g., patient registries), published case studies or annual summary reports prepared by licence holders. All adverse reaction reports are subjected to the same rigorous assessment process, irrespective of the reporting source.

## Outcomes

### Type of adverse reaction experienced

Per the *Cannabis Regulations* (2019), an adverse reaction is defined as “a noxious and unintended response to cannabis or a cannabis accessory that contains cannabis”. An adverse reaction can be serious, non-serious or unknown (i.e., no information provided on seriousness of reaction). A serious adverse reaction is defined in the *Cannabis Regulations *as “an adverse reaction that requires inpatient hospitalization or a prolongation of existing hospitalization, causes congenital malformation, results in persistent or significant disability or incapacity, is life-threatening or results in death”. This regulatory definition is further expanded in Health Canada reporting guidance to include ‘other medically important condition’, which includes events that are not immediately life threatening or result in death or hospitalization but may jeopardize the patient or may require a medical intervention (e.g., ambulatory services, emergency department visits, outpatient visits with a health care practitioner or at-home medical interventions) to prevent a serious outcome (2022). In addition, reporters can select several options for reason for seriousness when submitting a case report. Therefore, reporting form options are listed as:

- caused/prolonged in-patient hospitalization;
- disability/incapacity;
- congenital abnormality/birth defect;
- needed medical attention/other medically important condition;
- life-threatening; and/or
- death.

Seriousness and reason for seriousness are presented as originally reported to the Canada Vigilance database.

### Reason for use

Reason for use categorizes an individual’s use of cannabis into medical or non-medical use. Cannabis use for medical purposes includes cases described as involving the cannabis being used pursuant to a medical authorization document provided by a health care practitioner or cases that report the use of the cannabis for a medical or therapeutic purpose, without mention of a medical authorization document. Cases are classified as involving cannabis used for non-medical purposes when the report indicates non-medical use, or when no information is provided for reason for use or a possible therapeutic indication. As such, reason for use is a created outcome based on the verbatim of the case report submitted to the Canada Vigilance database, and is self-reported.

### Reason for medical use (indication)

Upon case report submission, reporters are asked to include an indication for use regarding the suspected cannabis product. This is a free text field that allows the reporter to include multiple indications. Responses have been manually grouped into broader categories for reporting purposes (e.g., sleep aid, pain, recreational, etc.).

### Reported event(s)

All events cited in a case report are coded by trained personnel at Health Canada using the Medical Dictionary for Regulatory Activities (MedDRA®), an international set of standardized medical terms for symptoms, signs, diseases, syndromes and diagnoses. There are five levels to the MedDRA® hierarchy, including ‘System Organ Class (SOC)’,'High Level Group Term (HLGT)’, ‘High Level Term (HLT)’, ‘Preferred Term (PT)’ and ‘Lowest Level Term (LLT)’. Events in this analysis are presented by SOC and PT as these categories strike the best balance between clarity, consistency, and usability (i.e., clinically meaningful and standardized), supporting both detailed and aggregate analysis, aligning with regulatory expectations and improving data quality. HLGTs and HLTs were not selected as they are too broad for detailed reporting and too narrow for high-level summaries, while LLTs were too granular and inconsistent for standardized reporting.

### Cannabis product(s) involved

Reporters are encouraged to include as much detail as possible regarding the suspected cannabis product in their adverse reaction case report, including product name, the name of the licenced producer and the product lot number. With this information, Health Canada can gather additional information on the product that allows for further classification, including product class and cannabinoid dominance (i.e., delta-9-tetrahydrocannabinol [THC] predominant, cannabidiol [CBD] predominant, balanced, unknown). The threshold for determining predominance is a cannabinoid ratio of 1.2:1 or greater (e.g., THC predominant is considered a THC a CBD ratio greater than 1.2:1). A ratio between 1.2:1 and 1:1.2 is considered balanced. Each suspected cannabis product within a case is assigned a cannabinoid dominance based on available product information, therefore, there may be multiple suspected products listed for a case (i.e., the total number of suspected products may exceed the total number of cases). Further, a phased approach was taken for legalization and regulation of cannabis for non-medical purposes. As such, only dried cannabis, fresh cannabis and ingestible oils were legally available in 2018 (phase 1). Extracts for inhalation (e.g., vapes), edibles, topicals and other product classes entered the market place at the end of 2019 (phase 2).

### Causality

The inclusion of an adverse reaction case report in the database does not necessarily mean that there is a causal relationship between the reported cannabis product(s) and the reported event(s). Additional scientific investigations are required to establish a cause-and-effect relationship. Health Canada conducts causality assessment for each individual event within a case report that is reported as serious to Health Canada, is deemed to be medically important or is suspected of being serious (where no indication of seriousness is provided). Events from non-serious case reports are not assessed for causality because these cases often report milder, and often known, adverse reactions and, therefore, are lower priority. Serious cases are more likely to have significant public health implications. Assessing causality in these instances helps identify potential risks in a timely manner and informs risk mitigation measures to protect public health. This assessment is conducted by trained Regulatory Affairs Specialists and is based primarily on the World Health Organization-Uppsala Monitoring Centre’s (WHO-UMC) system for standardized case causality assessment (2024). There are six WHO-UMC causality categories:

- ‘Certain’, which requires a stringent level of evidence, including both laboratory and medical confirmation, and lacks alternative explanation;
- ‘Probably/Likely’, where there is sufficient information to judge that the cannabis product probably contributed to the adverse reaction and the contribution of other factors was considered to be unlikely;
- ‘Possible’, where there is a reasonable possibility that the cannabis product may have contributed to the adverse reaction, but the contribution of other factors cannot be ruled out (for example, concomitant medications, co-morbidities, etc.);
- ‘ Unlikely’, where the cannabis product is judged to not play a causative role in the reported adverse reaction due to the presence of other probable factors;
- ‘Conditional/Unclassified’, where more information is required to make a definitive conclusion; and
- ‘Unassessable/Unclassifiable’, where there is insufficient information to establish a causal association between the cannabis product and the reported adverse reaction.

Causality assessment is a clinical investigation of cases and is a routine practice in pharmacovigilance to determine the likelihood of association between the product(s) and the adverse event(s). This practice supports the identification of potential safety concerns that may require further investigation or actions by the regulatory authority (e.g. Health Canada) and/or the licence holder. The causality of each event is based on the information reported in the case and through follow-up where possible. Cases may lack sufficient information to assess the causal association between event(s) and exposure to the cannabis products; in these cases, causality is not attributed to the cannabis product (i.e., “unassessable”).

#### Data analysis

A descriptive analysis of all adverse reaction cases involving legal cannabis products in a suspected role received by Health Canada from October 17, 2018, to December 31, 2024, was conducted to better characterize report case patterns, including the demographic profiles and cannabis use patterns of individuals reporting adverse reactions.

For all cannabis adverse reaction cases, sex (i.e., male, female, not reported), seriousness (i.e., serious, non-serious, seriousness unknown), reason for seriousness (i.e., death, like-threatening, hospitalization, disability, congenital malformation, other), reason for use (i.e., for medical or non-medical purposes), and indication for use were extracted and analyzed. Age was extracted, aggregated and presented as a mean ± standard deviation, median and range. Adverse event frequencies were analyzed by MedDRA® SOC and MedDRA® PT, by calendar year. Of note, ‘2018’ only covers the period of October 17, 2018, to December 31, 2018, in this study.

Year-over-year proportional changes case characteristics were observed and described; however, no formal statistical tests of significance were conducted. Given the nature of spontaneous reporting data, statistical testing is not appropriate due to several limitations, including the lack of a known denominator population, the presence of underreporting and other reporting biases, and the absence of a controlled comparison group.

Causality, as assessed by Health Canada, was presented for all cases submitted in 2022, 2023 and 2024; prior years were excluded in this portion of the analysis due to differences in assessment processes impacting comparability. Total number of cases, cannabis product class and cannabinoid dominance were also analyzed by submission calendar year.

## Results

**Fig. 1:**
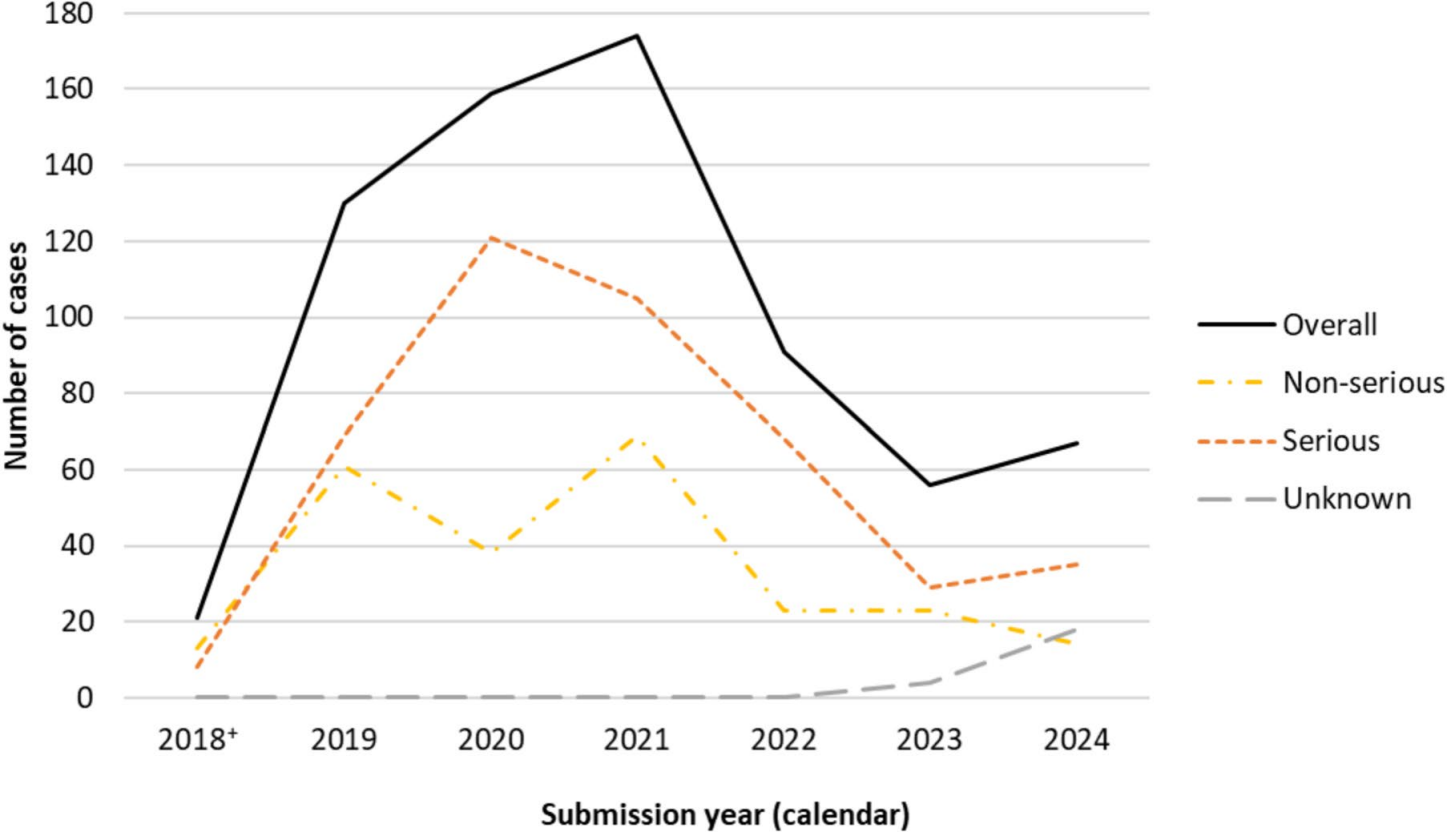
Number of adverse reaction cases involving cannabis products submitted to Health Canada by seriousness and calendar year of submission, 2018–2024. *Note.* This figure includes cases where seriousness was unknown. In 2023, Health Canada added this new category to capture cases submitted using alternate reporting forms that may not provide the option to identify seriousness. As a result, these cases may be captured differently in other databases

A total of 698 unique adverse reaction cases involving at least one legal cannabis product in a suspected role were submitted to Health Canada from October 17, 2018, to December 31, 2024. The number of cases submitted overall peaked in 2021 and have generally declined since, with a slight increase in 2024 (Fig. 1). Most cases, year-over-year, were reported as serious and were primarily submitted by cannabis licence holders who have a regulatory obligation to submit any serious adverse reactions they become aware of (data not shown).

## Case demographics

Table 1 shows demographic and use characteristics for individuals who reported experiencing an adverse reaction with a legal cannabis product in the reference period. The mean age of individuals experiencing adverse reactions to cannabis was 56.0 years (standard deviation 20.0 years), with a range of 5 months to 99 years. Females were involved in 45.4% (n = 317/698) of all cases versus 35.8% (n = 250/698) of cases involving males. In the remainder of cases (n = 131), the sex of the individual was not reported. Cases were often reported as serious (62.3%, n = 435/698) with ‘other medically important condition’ as the primary reason for seriousness (58.6%, n = 255/435). Almost a third of serious cases involved hospitalization (32.0%, n = 135/435). Most individuals reported using cannabis for medical purposes (67.4%, n = 471/698). The most common medical use cited among medical consumers was pain management (20.6%, n = 97/471); 9.1% (n = 43/471) reported multiple indications for use.

**Table 1:** Demographic and other characteristics of individuals reporting an adverse reaction to cannabis by report seriousness†, 2018–2024

	**Serious**	**Non-serious**	**Total^**
Total (N)	435	241	698
Age (years)			
Mean (standard deviation)	58.3 (19.9)	50.6 (19.2)	56.0 (20.0)
Median	61.0	49.5	58.0
Range	5 months – 99 years	17 years – 88 years	5 months – 99 years
Sex, % (n)			
Female	55.4 (241)	31.1 (75)	45.0 (317)
Male	34.7 (151)	39.4 (95)	36.0 (250)
Unknown	9.9 (43)	29.5 (71)	19.0 (131)
Reason for use, % (n)			
Medical	80.2 (349)	46.5 (112)	67.5 (471)
Non-medical	19.8 (86)	53.5 (129)	32.5 (227)
Reason for seriousness, % (n)			
Death	0.7 (3)	0.0 (0)	0.4 (3)
Disability	1.6 (7)	0.0 (0)	1.0 (7)
Hospitalization	32.0 (139)	0.4 (1)ǂ	20.1 (140)
Life-threatening	5.5 (24)	0.0 (0)	3.4 (24)
Multiple reasons reported	1.6 (7)	0.0 (0)	1.0 (7)
Not reported as serious	Not applicable	98.8 (238)	34.1 (238)
Other medically important	58.6 (255)	0.8 (2)	36.8 (257)
Unknown	Not applicable	Not applicable	3.2 (22)
Indication for medical use, % (n)			
Appetite disorders	0.7 (3)	0.0 (0)	0.4 (3)
Auto-immune disorders	0.7 (3)	0.4 (1)	0.6 (4)
Developmental disorders	0.7 (3)	0.0 (0)	0.4 (3)
Mental health disorders	3.0 (13)	0.8 (2)	2.1 (15)
Multiple reasons	9.9 (43)	3.3 (8)	7.6 (53)
Neurological disorders	2.1 (9)	0.8 (2)	1.6 (11)
Other	0.7 (3)	0.0 (0)	0.4 (3)
Pain management	22.3 (97)	6.6 (16)	16.2 (113)
Recreational use	6.0 (26)	11.2 (27)	8.2 (57)
Sleep issues	7.6 (33)	0.4 (1)	5.2 (36)
Unknown	37.5 (163)	75.1 (181)	6.6 (46)
Unknown medical use*	9.0 (39)	1.2 (3)	6.6 (46)

^†^ As reported in the case report

^ Includes cases where the seriousness of the report was not indicated (i.e., “seriousness unknown”)

ǂ In some cases, Health Canada may determine that a non-serious adverse reaction (as reported) meets the criteria for a serious adverse reaction based on details provided in the verbatim of a report or if additional follow up is received. In these cases, the case report is upgraded to “serious”. This is why the value exhibited in this line differs slightly from the number of non-serious reports included under “seriousness of report”

^*^ “Unknown medical use” is applied where the reporter indicates use for medical purposes, or that an authorization for medical use was obtained, but a specific indication was not provided

In terms of differences observed between serious and non-serious cases, individuals involved in serious cases were older, with a mean age of 58.3 years (standard deviation 19.9 years), female (55.4%, *n* = 241/435) and using cannabis for self-reported medical purposes (80.2%, *n* = 349/435). Comparatively, individuals involved in non-serious cases were slightly younger with a mean age of 50.6 years (standard deviation 19.2 years), male (39.4%, *n* = 95/241) and using cannabis for non-medical purposes (53.5%, *n* = 129/241).

These observations were generally consistent year-over-year (data not shown).

## Adverse events

Table 2 shows the count of adverse events (MedDRA® System Organ Class [SOC]) that were reported in cases involving legal cannabis products and were included in this analysis by submission calendar year. Overall, there were 27 distinct SOCs represented, with 2,486 total MedDRA® Preferred Term (PT) events reported (average: 3.6 events per case; range: 1 to 28 events). The average number of events reported per case initially decreased from 2018 to 2019 and remained relatively stable thereafter before increasing again in 2024 (2018: 4.6; 2019: 3.4; 2020: 3.5; 2021: 3.6; 2022: 3.2; 2023: 3.7; 2024: 4.0). In general, the top SOCs year-over-year remained stable. The top SOC overall was psychiatric disorders with 439 events reported, followed by nervous system disorders (n = 411).

**Table 2:** Count of adverse events‡ (MedDRA® SOC) to cannabis reported in adverse reaction cases, 2018–2024

MedDRA® SOC	Submission year (calendar)							Total
	2018	2019	2020	2021	2022	2023	2024	
**Blood and lymphatic system disorders**	0	1	1	0	2	0	0	4
**Cardiac disorders**	2	9	7	11	7	6	7	49
**Congenital, familial and genetic disorders**	0	1	4	0	0	0	1	6
**Ear and labyrinth disorders**	2	4	7	8	1	4	3	29
**Endocrine disorders**	1	0	0	0	0	0	0	1
**Eye disorders**	2	8	8	8	4	6	9	45
**Gastrointestinal disorders**	4	55	70	78	40	30	29	306
**General disorders and administration site conditions**	9	63	85	94	47	22	32	352
**Hepatobiliary disorders**	0	3	0	1	0	0	0	4
**Immune system disorders**	3	7	9	3	2	4	4	32
**Infections and infestations**	2	7	8	10	5	3	5	40
**Injury, poisoning and procedural complications**	0	23	33	30	14	3	5	108
**Investigations**	3	18	14	20	5	4	3	67
**Metabolism and nutrition disorders**	0	4	4	8	2	1	0	19
**Musculoskeletal and connective tissue disorders**	2	15	6	9	4	1	2	39
**Neoplasms benign, malignant and unspecified (incl cysts and polyps)**	0	2	1	3	4	0	5	15
**Nervous system disorders**	13	88	84	88	43	48	47	411
**Pregnancy, puerperium and perinatal conditions**	0	0	1	0	0	0	0	1
**Product issues**	6	20	17	25	19	5	8	100
**Psychiatric disorders**	20	68	130	102	45	36	38	439
**Renal and urinary disorders**	0	3	4	2	1	1	0	11
**Reproductive system and breast disorders**	0	1	2	0	0	0	2	5
**Respiratory, thoracic and mediastinal disorders**	14	19	43	82	35	22	44	259
**Skin and subcutaneous tissue disorders**	8	21	13	23	8	8	12	93
**Social circumstances**	0	0	1	1	0	0	2	4
**Surgical and medical procedures**	1	2	3	2	3	0	2	13
**Vascular disorders**	4	3	5	9	3	5	5	34

^‡^ Multiple SOCs may exist within one individual case

At the MedDRA® PT level, the most frequently reported events overall included hallucination (n = 119), headache (n = 81), nausea (n = 73), dizziness (n = 57) and dyspnoea (n = 51). Among serious cases, the most frequently reported PTs included hallucination (n = 110), dyspnoea (n = 37), dizziness (n = 37), nausea (n = 33) and diarrhoea (n = 30). Among non-serious cases, the most frequently reported PTs included headache (n = 55), nausea (n = 40), throat irritation (n = 29), malaise (n = 25) and oropharyngeal pain (n = 24).

**Fig. 2:**
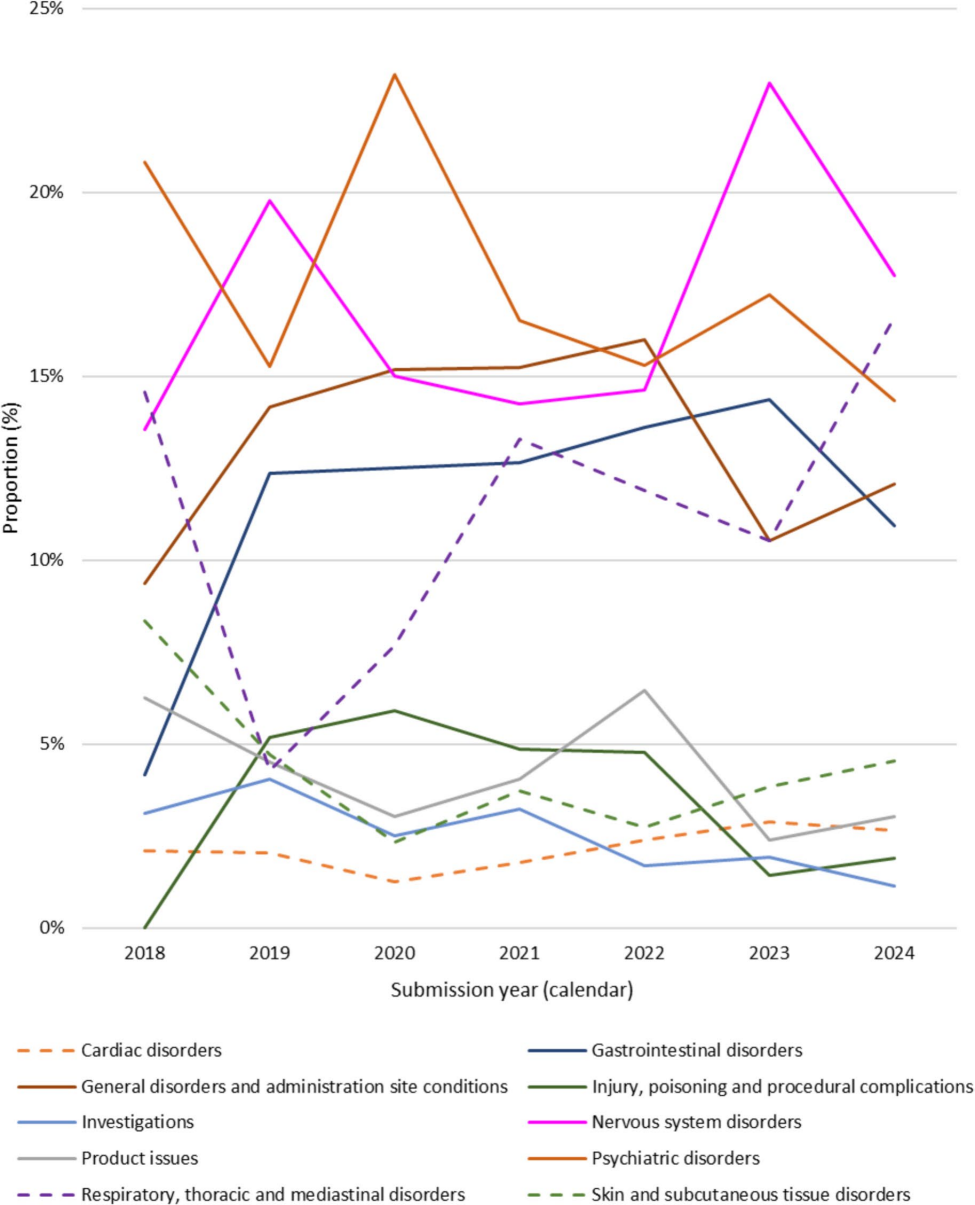
Change in the proportion (%) of the top 10 MedDRA® SOCs reported in adverse reaction cases by calendar year of submission**,** 2018–2024

Figure 2 shows the change in the proportion of the top 10 SOCs over time. Of particular interest is general disorders and administration site conditions (n = 352) which includes drug interactions, drug ineffective, condition aggravated, etc. The proportion of event attributed to this SOC increased from 9.4% (n = 9/96) in 2018 to 16.0% (n = 47/294) in 2022 and proportion of these events has remained comparatively high since, despite a decrease in the proportion of these cases in 2024, to 12.1% (n = 32/265). Variability is present in the proportion of events attributed to respiratory, thoracic and mediastinal disorders (n = 259) year-over year, despite a consistent decline in the prevalence of dried flower use over the same period per the Canadian Cannabis Survey (2024). However, an overall increase in events attributed to respiratory, thoracic and mediastinal disorders was observed from 14.6% (n = 14/96) 2018 to 16.6% (n = 44/265) in 2024. Events captured under this category may be attributed to the route of administration of the cannabis product, namely inhalation, and not necessarily the cannabis itself (e.g., throat irritation, oropharyngeal pain, cough, respiratory tract irritation, etc.). In most cases, these events resulted in non-serious outcomes.

An upward trend, particularly in later years, was generally observed in the relative proportion of events attributable to the following SOCs: cardiac disorders, eye disorders, gastrointestinal disorders, nervous system disorders, psychiatric disorders, skin and subcutaneous tissue disorders, and vascular disorders. Also of interest is the SOC injury, poisoning and procedural complications (n = 108) which includes cases of poisoning, accidental exposures, product selection error and package/label confusion among others. The relative proportion of events attributable to this SOC has declined since its peak in 2020 (from 5.9% (n = 33/560) in 2020 to 1.9% (n = 5/265) in 2024).

## Cannabis products involved in adverse reaction reports

Table 3 shows the characteristics of cannabis products involved in adverse reaction cases, as reported in the case report. At the end of 2018, the most frequently reported cannabis product class involved in adverse reaction cases was dried cannabis. This shifted to cannabis extracts, specifically ingestible oils and softgel capsules, in 2019 and onward. As a reminder, only dried cannabis, fresh cannabis and ingestible oils were legally available in 2018. Extracts for inhalation, edibles, topical and other product classes entered the market at the end of 2019. Individuals involved in cases with cannabis extracts were often female (53.8%, n = 258/480) and older in age (65 + : 38.3%, n = 184/480). Conversely, individuals involved in cases with dried cannabis were often male (42.9%, n = 78/182) and young-middle aged (18–64: 46.7%, n = 85/182).

**Table 3:** Characteristics of cannabis products involved in adverse reaction cases, 2018–2024

	**Submission year (calendar)**							**Total**
	2018	2019	2020	2021	2022	2023	2024	
Product class								
Extract	8	88	136	129	48	36	35	480
Dried	13	41	20	37	32	16	23	182
Edible	0	0	2	5	8	2	6	23
Topical	0	0	0	2	3	2	0	7
Multiple	0	1	1	0	0	0	3	5
Unknown	0	0	0	1	0	0	0	1
Cannabinoid dominance								
THC predominant	16	44	45	80	46	28	37	297
CBD predominant	1	62	71	53	29	20	16	252
Balanced ◊	2	6	11	22	10	2	4	57
Multiple ǂ	0	6	17	12	5	4	8	51
Unknown	2	12	15	7	1	2	2	41

◊ THC:CBD ratio between 1.2:1 and 1:1.2

ǂ Multiple suspected cannabis products with various cannabinoid dominances reported

Also shown in Table 3, most involved cannabis products with a THC to CBD ratio of > 1.2 (i.e., THC predominant) (*n* = 297). However, cases involving CBD predominant cannabis products (i.e., CBD to THC ratio of > 1.2) were relatively more common in 2019 and 2020. Across all years, cases involving balanced products were uncommon. In terms of outcome severity, 28.1% of cases involving only balanced cannabis products (16/57) required hospitalization compared to 18.9% of cases involving only THC predominant cannabis products (56/297) and 18.3% of cases involving only CBD predominant cannabis products (46/252). Cases involving multiple products reported requiring hospitalization in 25.5% of cases (13/51).

In addition, the number of cases involving multiple different product classes (e.g., a dried cannabis product and a cannabis extract product) has also remained low despite an increase in the availability of different cannabis products over the years, particularly following amendments to the *Cannabis Regulations* in 2019. There were 823 products reported across 698 adverse reaction cases. The average number of suspect cannabis products involved in case reports has remained stable from 1.3 products per case in 2018 to 1.3 products per case in 2024; the average overall was 1.2 products per case.

**Fig. 3:**
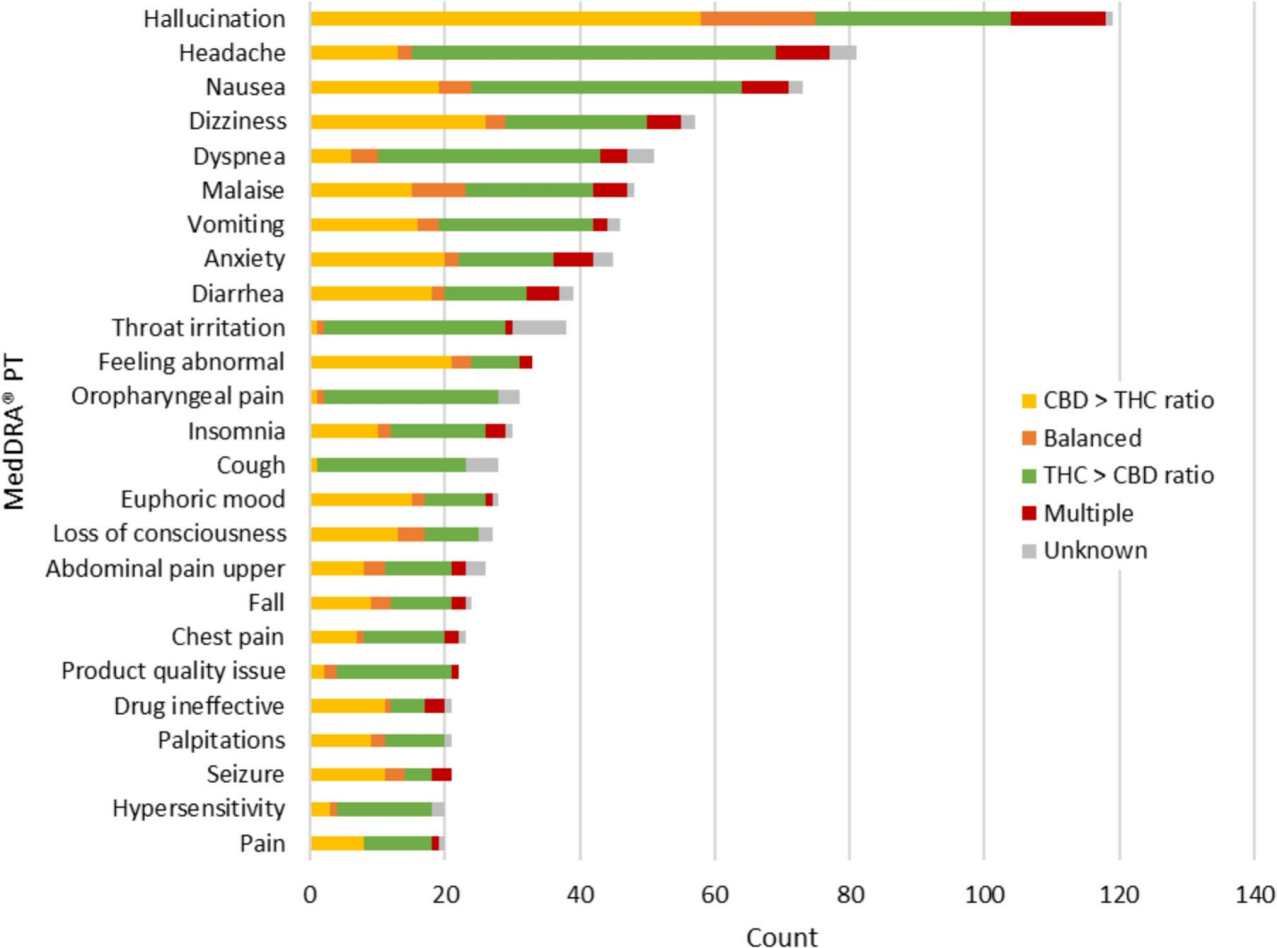
Frequency of top 25 adverse events‡ (MedDRA® PT) overall as reported in adverse reaction cases by cannabinoid ratio, 2018–2024

The type of events reported varied by cannabinoid dominance of the suspected cannabis product (Fig. 3). For example, hallucination, dizziness, anxiety, diarrhea, feeling abnormal, euphoric mood, loss of consciousness, seizure and ‘drug ineffective’ were more frequently reported among cases involving cannabis products with a greater ratio of CBD to THC. Conversely, headache, nausea, dyspnea, throat irritation, oropharyngeal pain, insomnia, cough, upper abdominal pain, chest pain and hypersensitivity were more commonly reported with cannabis products containing a greater ratio of THC to CBD. These observations are based on the information reported with the case report and are not reflective of a causal association.

## Causal association

Table 4 shows the causality assignment for each event assessed in serious adverse reaction cases only (668 events reported across 176 cases). Most events were assessed as being possibly related to the cannabis based on the information made available in the case report and through follow up with the reporter. ‘Possible’ causality is assigned where there is a reasonable possibility that the cannabis product may have contributed to the adverse reaction, but the contribution of other factors could not be ruled out (e.g., concomitant medications, co-morbidities, etc.). A small number of events were assessed as being certainly related to the cannabis (n = 7), where there was laboratory and/or medical confirmation and no other alternative explanation.

**Table 4:** Causality assessment of events involved in serious* adverse reaction cases, 2022–2024

Assessed causality	Number of events
Certain	7
Probable	74
Possible	354
Unlikely	97
Unassessable	136

^*^ This table presents causality for events involved in serious adverse reaction cases. Causality for non-serious cases is sometimes assessed, but is not presented in this summary

Notably, in all cases involving a fatal outcome (n = 3), the fatal outcome was assessed as unlikely to be related to the cannabis product. All three fatal cases involved individuals with complicated medical histories and use of multiple concomitant medications, in addition to other alternative explanations and/or cause of death being identified (e.g., via autopsy).

**Fig. 4:**
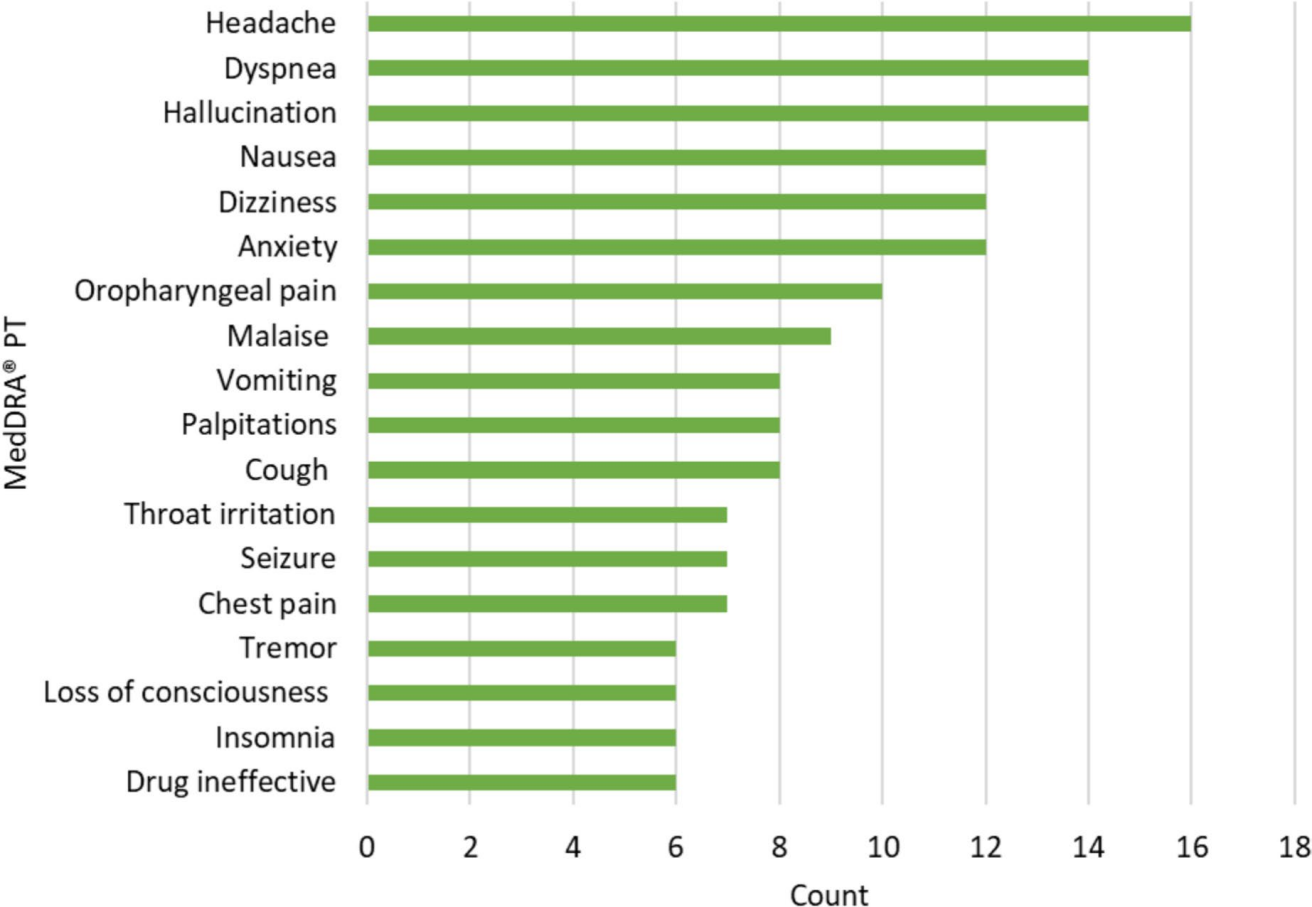
Most frequent adverse events‡ (MedDRA® PT) reported in serious adverse reaction cases where causality was assessed as certain, probable or possible, 2022–2024

Figure 4 shows the most frequently reported adverse events by MedDRA® PT where causality was assessed by Health Canada as being certainly, probably or possibly related to the suspected cannabis product(s), based on the available information (435 events across 124 cases). The most frequently reported event was headache (n = 16), followed by dyspnea (n = 14) and hallucination (n = 14).

## Discussion

This is the first publication examining trends in adverse reactions involving legal retail cannabis products in Canada following the legalization and regulation of cannabis. This study revealed that since legalization, the number of cannabis adverse reaction cases received by Health Canada initially increased from 2018 to 2021 and declined thereafter, with a slight increase in 2024. This pattern was observed for serious adverse reaction cases, and the number of cases involving use of cannabis for medical purposes. However, this decline in cannabis adverse reaction reporting should not be interpreted as meaning that cannabis products are now more safe or less likely to result in adverse reactions. It’s possible that, like adverse reaction reporting for pharmaceuticals and health products, cannabis may exhibit the Weber Effect whereby the number of reports for pharmaceuticals and health products exhibits an initial peak within the first 2 years of product approval and then experiences a rapid decline in reporting thereafter (2015). This aligns with observations regarding the submission of cannabis-related adverse reaction reports; a sharp increase in the first 2 full years following legalization, followed by a rapid decline, particularly for serious adverse reaction reports.

Other possible reasons for this decline in reporting may include a general unawareness of reporting obligations for mandatory reporters (i.e., licence holders) and an unawareness (2011) about the opportunity to report to Health Canada, especially for voluntary reporters (e.g., physicians, consumers) who are not obligated to report adverse reactions to Health Canada. According to the 2024 Canadian Cannabis Survey, only 22.3% of respondents were aware of the ability to report adverse reactions from cannabis to Health Canada (2024). Underreporting is also a common issue. Among 2024 Canadian Cannabis Survey respondents who reported experiencing an adverse reaction and were aware of the ability to report, only 5% reported the adverse reaction to Health Canada (2024). There may also be confusion about what types of reactions should be submitted to Health Canada, an unwillingness to report, and/or issues related to the report submission process that impede reporting (e.g., lengthy forms, inconvenient submission methods, etc.). This has been noted as a common bias in spontaneous reporting systems in which reporters, particularly health professionals, tend to report rarer and more serious adverse reactions (2024). For example, individuals may not feel it necessary to submit reactions to cannabis that they consider ‘expected’. Our study also found that certain events were more frequently reported with products containing a greater CBD to THC ratio versus products with a greater THC to CBD ratio. Individuals may consider some of these events to be ‘expected’, particularly with THC consumption. However, the concept of ‘expectedness’ in reference to the effect of a substance, as referenced for pharmaceuticals and health products regulated under the *Food and Drugs Act* (2023) and its regulations, does not exist under the*Cannabis Act* and its regulations, therefore, Health Canada considers all adverse reactions to cannabis to be unexpected. It is likely that some individuals might not be aware of this distinction between these frameworks which could lead to a decision to not report the adverse reaction.

Overall, individuals experiencing cannabis-related adverse reactions were generally older and female. The observed sex disparity aligns with previous research which has identified a tendency for females to report adverse drug reactions more than males (2019, 2019). Cases also often involved use of cannabis for self-reported medical purposes, with pain being the most frequently reported use of cannabis for medical purposes (i.e., indication). Cannabis extracts, particularly ingestible oils, were the most common type of suspected cannabis product. Notably, adults over 55 years of age may be more sensitive to cannabis and have a higher risk of experiencing adverse reactions, especially if they have certain medical conditions or use other health products, which may increase the risk of possible interactions. It’s also important to note that Health Canada only started receiving reports involving inhaled extracts, cannabis edibles and topicals in 2020 and 2021 respectively, aligning with the implementation of amendments to the*Cannabis Regulations* allowing for the sale of these product classes in the Canadian marketplace. However, adverse reaction cases reported to Health Canada involving legal cannabis edibles and topicals have remained low.

The most frequently reported events were generally consistent year-over-year. Factors that may contribute to the experience of these events include: dosage; route of administration; format and potency of cannabis; prior exposure to cannabis (e.g., cannabis naïve consumers); and the age and health status of patients including pre-existing health conditions and use of concomitant medications (2022). Reporting of cannabis adverse reactions may also be impacted by motivation to report, risk tolerance or awareness of reporting, and knowledge or awareness of effects of cannabis and cannabinoids. Of particular interest is the overall decline in the relative proportion of events attributable to the SOC ‘injury, poisoning and procedural complications’ which includes cases of poisoning, accidental exposures, product selection error and package/label confusion, etc. The*Cannabis Act* includes several requirements related to packaging, labelling and promotion of cannabis products to ensure that such products are less likely to appeal to youth, reduce the risk of accidental consumption and inform consumers of the potential risks associated with cannabis use. Health Canada continues to monitor reports involving these types of events and mitigate these risks as necessary.

A well-known challenge of spontaneous reporting systems is the limited case detail making it difficult to determine causality between the substance and adverse event (2002, 2013). While there are limitations with report completeness and quality inherent to the vigilance framework for cannabis products, there is an opportunity to follow up with reporters, collecting additional information that allows Health Canada to assess causality for each report. However, if information is missing and follow up is not possible, this may result in a case being categorised as ‘unassessable’. In the current study, most of the events that underwent assessment were assessed as being “possibly” associated with the reported cannabis product.

### Limitations

There are several limitations to consider with this study and data. First, adverse reaction reports are often spontaneously reported to Health Canada and cannot be used to determine the incidence or prevalence of adverse reactions to cannabis in the general population. Second, serious adverse reaction reports have a greater representation in this dataset as licence holders have a regulatory obligation to report these to Health Canada under paragraph 248.1(1)(a) of the *Cannabis Regulations*. The submission of non-serious adverse reactions by licence holders to Health Canada as individual case reports is voluntary; therefore, these cases are likely underreported and underrepresented in this dataset. Third, reporting of adverse reactions by consumers, health professionals, hospitals, medical cannabis clinics and provincial and territorial retailers is voluntary for cannabis products, therefore, both serious and non-serious cases from these sources are likely underreported. Fourth, individuals experiencing serious outcomes or using cannabis products for medical purposes may be more motivated to report adverse reactions or seek out medical attention. Fifth, several factors may influence the number and quality of adverse reaction reports submitted to Health Canada such as: consumer medical history; reason for cannabis use; length of time a product is on the market; media coverage; awareness, motivation and ability to report; stigma surrounding use of cannabis; and nature of reports (e.g., spontaneous reports versus studies or other organized data-collection systems). For example, Health Canada continues to raise awareness about reporting of cannabis adverse reactions to a variety of external partners and collaborators, potentially impacting the number and quality of reports being received. As such, an increase in reporting, overall or for specific events, may be more reflective of effective outreach and not necessarily a change in the risk profile of legal cannabis products. Finally, this study presented trends across 6 full years and 1 partial year of data in which regulatory amendments have occurred. Long-term data are essential to verify the consistency and reliability of the patterns observed in the current study.

## Conclusions

The vigilance framework for cannabis established when the *Cannabis Act* came into force has been an essential tool for the ongoing collection of risk information about cannabis products available for purchase in the legal Canadian marketplace. Six years into legalization and regulation of cannabis, these data have helped Health Canada to identify previously unrecognized side effects; contributed to international data about risks of cannabis products available for sale in Canada; and guided communications related to new and emerging risks. Health Canada continues to monitor this source of real-world data, leveraging it to make informed regulatory decisions. In addition, the observed decline in case submissions in recent years highlights the need to enhance adverse reaction reporting for cannabis through various means.

## Data Availability

Most of the data are publicly available at https://www.canada.ca/en/health-canada/services/drugs-health-products/medeffect-canada/adverse-reaction-database.html. Annual aggregate summaries of cannabis adverse reaction data are also available at https://www.canada.ca/en/health-canada/services/publications/drugs-health-products/data-cannabis-adverse-reactions-reports-overview.html.

## Abbreviations

CBD: Cannabidiol
HLGT: High Level Group Term
HLT: High Level Term
LLT: Lowest Level Term
MedDRA®: Medical Dictionary for Regulatory Activities
PT: Preferred Term
SOC: System Organ Class
THC: Delta-9-Tetrahydrocannabinol
WHO-UMC: World Health Organization-Uppsala Monitoring Centre
